# Combined Use of Electron and Light Microscopy Techniques Reveals False Secondary Shell Units in Megaloolithidae Eggshells

**DOI:** 10.1371/journal.pone.0153026

**Published:** 2016-05-04

**Authors:** Miguel Moreno-Azanza, Blanca Bauluz, José Ignacio Canudo, José Manuel Gasca, Fidel Torcida Fernández-Baldor

**Affiliations:** 1 Geobiotec, Departamento de Ciências da Terra, Faculdade de Ciências e Tecnologia, FCT, Universidade Nova de Lisboa, Caparica, Portugal; 2 Museu da Lourinhã, Lourinha, Portugal; 3 Grupo Aragosaurus–IUCA, Área de Paleontología, Facultad de Ciencias, Universidad de Zaragoza, Zaragoza, Spain; 4 Área de Mineralogía, Facultad de Ciencias, Universidad de Zaragoza, Zaragoza, Spain; 5 CONICET-Museo Olsacher, Zapala, Neuquén, Argentina; 6 Fidel Torcida Fernández-Baldor Colectivo Arqueológico-Paleontológico de Salas, Museo de Dinosaurios, Salas de los Infantes, Burgos, Spain; Perot Museum of Nature and Science, UNITED STATES

## Abstract

Abnormalities in the histo- and ultrastructure of the amniote eggshell are often related to diverse factors, such as ambient stress during egg formation, pathologies altering the physiology of the egg-laying females, or evolutionarily selected modifications of the eggshell structure that vary the physical properties of the egg, for example increasing its strength so as to avoid fracture during incubation. When dealing with fossil materials, all the above hypotheses are plausible, but a detailed taphonomical study has to be performed to rule out the possibility that secondary processes of recrystallization have occurred during fossilization. Traditional analyses, such as optical microscopy inspection and cathodoluminescence, have proven not to be enough to understand the taphonomic story of some eggshells. Recently, electron backscatter diffraction has been used, in combination with other techniques, to better understand the alteration of fossil eggshells. Here we present a combined study using scanning electron microscopy, optical microscopy, cathodoluminescence and electron backscatter diffraction of eggshell fragments assigned to *Megaloolithus* cf. *siruguei* from the Upper Cretaceous outcrops of the Cameros Basin. We focus our study on the presence of secondary shell units that mimic most aspects of the ultrastructure of the eggshell mammillae, but grow far from the inner surface of the eggshell. We call these structures extra-spherulites, describe their crystal structure and demonstrate their secondary origin. Our study has important implications for the interpretation of secondary shell units as biological or pathological structures. Thus, electron backscatter diffraction complements other microscope techniques as a useful tool for understanding taphonomical alterations in fossil eggshells.

## Introduction

Despite considerable differences in eggshell ultrastructure [1], dinosaur eggshells share a common formation process, which has been described for distantly related taxa such as ornithopods and theropods [2]. In this process, eggshell growth started after nucleation of radial calcite aggregates around the organic cores, which are sub-spherical aggregates of proteins and calcite microcrystals not always preserved after fossilization. These initial spheres, embedded in the eggshell membrane, acted as seeds for the cup-shaped bases of the mammillae, built of radial calcium carbonate crystals growing towards the outer surface of the eggshell [1,3,4]. The mammillae gradually converged, and the relations between the crystals and the protein net determine whether the individual aggregates are still identifiable within the outermost part of the eggshell (as in the shell units characteristic of the oofamily Megaloolithidae and to some extent the oofamilies Spheroolithidae and Fusioolithidae), or whether they become a continuous palisade of crystals (as in columns characteristic of most Prismatoolithidae and other theropod-related eggshells, including bird eggshells [1,5]).

**Fig 1:**
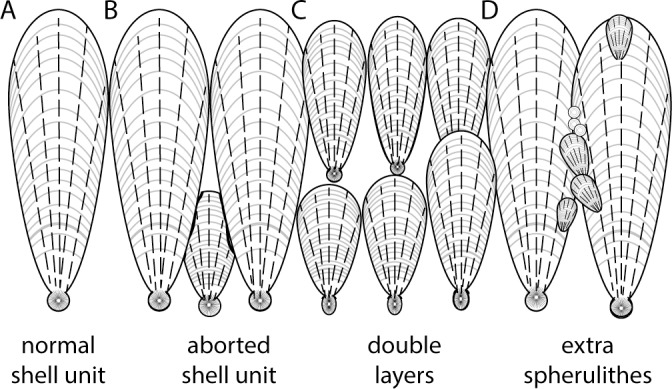
Schematic drawing of an idealized megaloolithid eggshell showing kinds of secondary shell units. (A) Normal shell unit. (B) Aborted shell units, which do not reach the top of the eggshell surface due to competition with neighboring units. Note that false aborted shell units have been previously reported to be caused by thin sections that cut the shell at an oblique angle. (C) Double layers that grow on top of the standard eggshell layer. (D) Extra-spherulites, which are smaller and nucleate at different points of the shell unit, with little or no relation with the rest of the eggshell.

The presence of secondary shell units–units that grow in other areas of the eggshell than the shell membrane–has been reported in modern eggshells of birds, turtles and crocodilians [6,7], as well as in several fossil oofamilies [7–10]. These secondary shell units are often seen as mammillary cones that start growing away from the inner surface of the eggshell [7]. Under optical microscopy, secondary shell units are almost indistinguishable from the true shell units and only their smaller size and abnormal location within the eggshell allow us to tell them apart from true shell units [11]. Three different types of secondary shell units can be differentiated (Fig 1): a) aborted shell units, that grow from the shell membrane but do not reach the outer surface, due to competition with neighboring shell units; b) double-layer shell units that grow on top of the normal shell units, forming a complete additional layer; and c) extra-spherulites, where small radiating shell-unit-like structures grow from the middle of the shell units [12]. The origin of these secondary shell units has been debated within the literature for decades [7,9–11,13–18]. Three main hypotheses have been postulated: 1) secondary shell units are pathological growths of the eggshell, sometimes caused by abnormally long stays of the egg within the oviduct that result in non-viable eggs [6,17]; 2) secondary shell units are taphonomic artifacts that grow during the fossil diagenesis of the eggshell material, caused by the precipitation of inorganic calcite around protein relics within the eggshell structure [11]; and 3) secondary shell units are original histostructural features of the eggshell, thus playing an important role in modifying the physical properties of the eggshells [8,10].

Here we present detailed crystallographic orientation and misorientation data from megaloolithid eggshells from the Late Cretaceous of Spain, obtained using electron backscatter diffraction (EBSD) and cathodoluminescence. The eggshells show abundant extra-spherulites throughout the eggshell structure. We falsify the hypothesis of a biological or pathological origin, and offer evidence for a taphonomic origin of this particular type of secondary shell unit. We then discuss the risk of considering these shell units as a source of data for taxonomic or paleobiological interpretations without appropriate characterization of their crystal structure and genesis.

### Geological Framework and Geographical Setting

**Fig 2:**
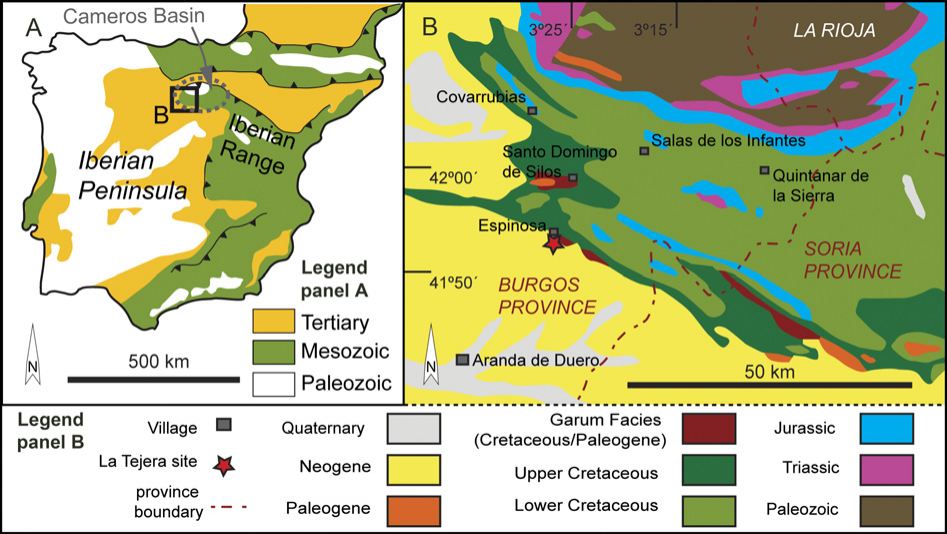
Geographical location and geological context of La Tejera site. (A) Simplified geological map of the Iberian Peninsula. (B) General map of the Cretaceous outcrops of the southwestern part of the Cameros Basin. Map redrawn from [25].

The eggshell fragments analyzed in this study come from La Tejera fossil locality, located one kilometer south of the municipality of Espinosa de Cervera, Burgos Province, northern Spain (Fig 2). Geologically, this locality is situated in the south flank of the Tejada anticline within the southwestern area of the Cameros Basin, the northwesternmost basin of the Mesozoic Iberian rift system (Fig 2). The Iberian basins were formed during the Late Jurassic to Early Cretaceous, and subsequently inverted during the Cenozoic alpine orogeny [19,20]. After the Early Cretaceous infill, a second post-rift phase took place during the Late Cretaceous, resulting in marine platforms extending due to a eustatic sea-level rise [21]. Late Cretaceous to Paleocene times corresponded to a period of relative tectonic quiescence and regional thermal subsidence of the Iberian basins [20].

La Tejera fossil site is located within the Santibañez del Val Formation (Lychnus limestone unit). These deposits are included within the Garumnian facies (i.e. a detritic, regressive succession around the Cretaceous-Paleogene boundary), which here overlies the shallow marine Santo Domingo de Silos Formation. The eggshell locality is Maastrichtian in age [22] whereas the Santibañez del Val Formation ranges from the Maastrichtian to the Eocene [23,24]. The eggshell-bearing unit consists of pink marls corresponding to shallow lacustrine deposits, which are interlocked with palustrine limestones. The marls contain quartz grains and abundant fossil fragments, including those of gastropods, ostracods and charophyte fructifications. Eggshell fragments are also common in the pink marls, but are not present in the limestones. For a detailed geological description of this locality see [22].

## Materials and Methods

The eggshell fragments were studied under a binocular microscope to select well-preserved specimens for thin section preparation. Five eggshell fragments were selected, embedded in epoxy resin and sliced into standard 30-μm thin sections, numbered MDS-TEC,215 to MDS-TEC,219. The remnant stubs of the thin section preparation were also numbered as MDS-TEC,215s to MDS-TEC,219s to allow future comparison with each corresponding thin section. The thin sections were studied and photographed using an Olympus BX 41 petrographic microscope. A preliminary paleoological study of the samples confirmed the affinities of the eggshell fragments, corroborating the identification provided by [22]. During this study, the presence of structures of uncertain origin was observed, here described as extra-spherulites. The most representative thin section, MDS-TEC,215, and the corresponding stub MDS-TEC,215s, were used in the analysis described below.

The thin section was mechanically polished using a 1-μm diamond abrasive. The superficial amorphous layer produced during mechanical polishing was removed using automated chemo-mechanical polishing with colloidal silica for 30 minutes [26]. The sample was coated with a thin layer of carbon for EBSD analysis [27]. Electron backscatter diffraction (EBSD) analyses were performed in the CamScan X500 CrystalProbe field-emission gun (FEG) SEM described by [28]. Electron backscatter diffraction patterns (EBSPs) were obtained using a 20-kV acceleration voltage, a 35-nA beam current, and a 25-mm working distance. They were automatically indexed and then analyzed using the software package CHANNEL 5, applying a grid spacing of 2 μm. At this resolution the thickness of the eggshell fragment (4 mm) presented a challenge, due to the long time required for the experiment. It was successfully analyzed in its entirety using 27 maps in a grid of 9 x 3. These maps were stitched together using the CHANNEL 5 software. The analysis lasted over 37 hours, and a small drift was observed between the maps obtained. However, this did not compromise our results. Raw EBSD data

Excluding large gaps where material was missing in the sample, excellent indexing was obtained for the full stitched map, so that only minimal post-acquisition data processing was necessary. After removal of “wild spikes” (isolated EBSD pixels that represent indexing errors), the data were improved by filling unresolved pixels with an orientation averaged over that of the closest six neighboring pixels. Data processing was carried out using selected datasets from band contrast values as a framework to avoid artifacts in grain size and shape [29]. This resulted in EBSD maps devoid of errors and artifacts and with a total of 87% indexed points. All-Euler maps, grain boundary maps and inverse pole figure coloring maps were used to present the data.

**Fig 3:**
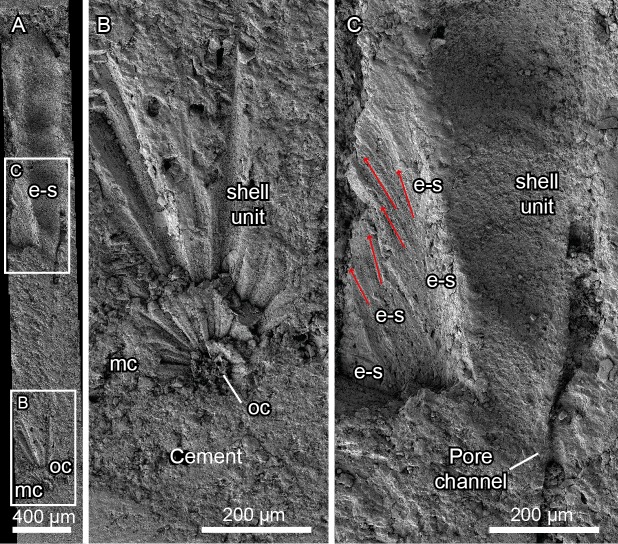
*Megaloolithus* cf. *siruguei*, PS-TEC,215, polished and etched radial thin section observed under scanning electron microscopy. (A) Panorama of fifteen SEM photographs covering the full height of a shell unit, including a preserved mammillary cone (mc), with location of areas figured in panels B and C. (B) Detail of the mammillae, showing the former organic core (oc) surrounded by calcite crystals with radial tabular ultrastructure. (C) Detail of the contact between a shell unit and an extra-spherulite (e-s). Red arrows show crystal orientation. Note the change in ultrastructure and the lack of an organic core in the extra-spherulite.

The stub containing the remaining eggshell fragment resulting from the thin section preparation of specimen PS-TEC,215 was polished with alumina and subsequently etched with pure potassium hydroxide (KOH) to remove the sedimentary matrix and impurities obscuring the eggshell structure [5]. A single pellet of KOH was placed on the polished surface and a drop of water was added, resulting in the dissolution of the scale and the etching of the eggshell. The stub was then cleaned using an ultrasound bath for 15 minutes. Etching of the remaining stub from PS-TEC,215 with KOH enhances the crystallographic features under secondary electrons (Fig 3). This preparation was studied using secondary electrons on a FESEM Carl Zeiss MERLIN^TM^ housed at the Servicio de Microscopía Electrónica de Materiales (Servicio de Apoyo a la Investigación, SAI, Universidad de Zaragoza), where images were acquired with a beam current of 3 kV and at a 15-mm working distance.

To test the hypothesis of a primary (biological or pathological) origin of the extra-spherulites, cathodoluminescence (CL) analyses were performed with a Nikon Eclipse 50i POL optical microscope coupled with a cathodoluminescence system (model CL8200 Mk5-1) at the Institut Català d’Arqueologia Clàssica (ICAC; Tarragona, Spain). Pictures were taken with a 15.1 kV beam current and 300 μA and 1 second of exposure, at F/4.6. The thin section was polished with 1-μm alumina for 10 seconds, enough to remove the carbon coating used for electron microscopy. Recent calcite amniote eggshells are non-luminescent under the above-mentioned cathodoluminescence observation conditions. This is due to the almost pure calcite composition of the mineralized phase of the eggshell, with no traces of cathodoluminescence inducer or quencher elements [30]. This is also the case with pristine dinosaur eggshells, which have shown almost no luminescence in several studies [11,31,32]. Also, and of more relevance for the present work, [33] assert that pristine specimens of *Megaloolithus siruguei* from the southern Pyrenees are non-luminescent. Finally, recrystallized specimens of megaloolithid-related eggshells have shown medium to bright luminescence [11,34].

## Results

                              Systematic Paleontology

                   Oofamily Megaloolithidae Zhao, 1979

                     Oogenus *Megaloolithus* Zhao, 1979

      Oospecies *Megaloolithus* cf. *siruguei* Vianey-Liaud, Mallan, Buscail and Montgelard, 1994

Figured material: an eggshell fragment, sliced in a 30-μm thin-section

Comments. Eggshell fragments from La Tejera locality were assigned by [22] to *Megaloolithus* cf. *siruguei* [12] on the basis of their isolated spherulitic shell units, compactituberculated ornamentation, tubocanaliculate pore system and shell thickness. This oospecies was previously reported from another locality within the Santibañez Formation (La Rosaca site [35,36]). Nevertheless, these authors consider that the pore system is different from the typical *M*. *siruguei* pore system, and that the thickness of the eggshells falls within the range of this oospecies. Five of the eggshell fragments included in the study by [22] are included in the present study. All the material described in the present publication is housed in the Dinosaur Museum of Salas de los Infantes (MDS; previously MPS), Salas de los Infantes, Burgos, Spain.

### Secondary electrons

Mammillary cones are formed by calcite crystals with radial tabular ultrastructure that radiate out of the organic core (Fig 3B). After a few microns, the limits or boundaries between crystals gradually fade, becoming indistinct in most of the shell unit. By contrast, extra-spherulites are built by aggregates of acicular crystals, which appear to be parallel in secondary electron images, but present a certain angle. Some of these acicular crystals bend gently at the top of the aggregates. They originate from anywhere within the shell unit, but are more frequent at the boundaries between shell units and present discrete boundaries with all the neighboring areas of the eggshell (Fig 3C). It is worth noting that, despite the abundance of extra-spherulites observed in thin sections (see below), they are hard to see in the prepared stub. This may be because of the reduced size of most extra-spherulites, which makes them harder to identify in SEM.

### Petrographic Microscopy

**Fig 4:**
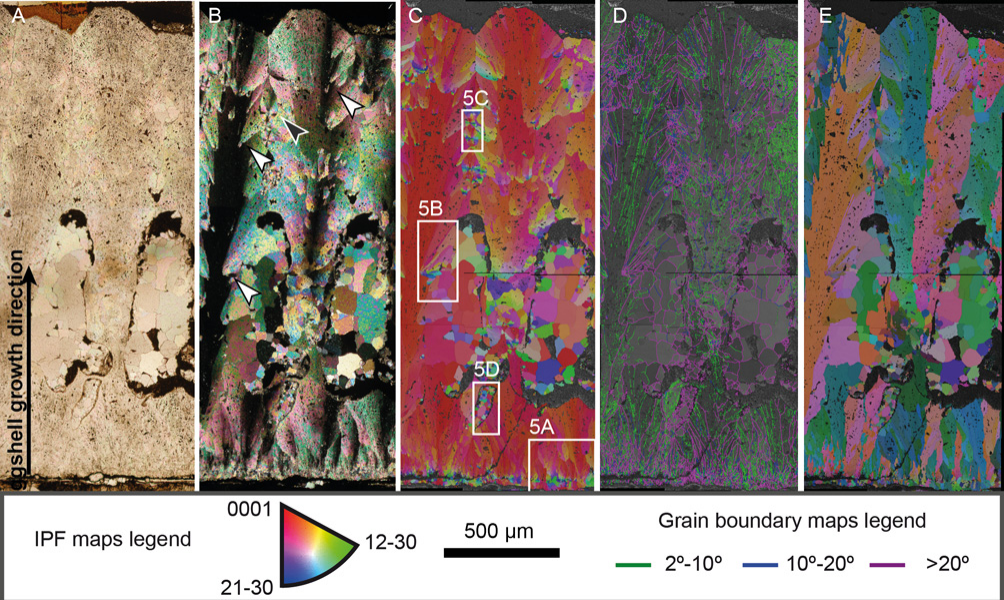
*Megaloolithus* cf. *siruguei*, PS-TEC,215, radial thin section. (A) Transmitted light microphotograph. The eggshell is built by tall shell units that grow for the full thickness of the eggshell. Note faint growth lines through all the eggshell thickness and large voids which may either be secondary dissolutions or part of a complex pore system. (B) Polarized light microphotograph. The general architecture of the eggshell, with undulating extinction partially obscured by the abundance of secondary shell units, which grow preferably in the margins of the true shell units. Spar cement fills the voids of the eggshell whereas a finer sized grain spar covers the inner surface at the bottom of the picture. (C) Inverse pole figure (IPFY1) coloring map showing the absolute orientation of the calcite crystals forming the shell units. At the base of the eggshell, the radial distribution of crystals quickly develops into a structure with preferred orientation. Most crystals of the shell units present red to orange coloration, indicating that the crystals grow with their c-axis oriented sub-parallel to the direction of eggshell growth. Extra-spherulites are built by fans of tabular calcite crystals with no relation to the true shell unit, and without disturbing the orientation of the crystals that form those units. (D) Grain boundary map over a band contrast image, showing the misorientation angles between crystals and sub-crystals in the eggshell structure. Note that the shell units present a profusion of low-angle boundaries (in green and blue) whereas secondary shell units are dominated by high-angle misorientation angles (in fuchsia). These high-angle boundaries are also seen in the spar cement that fills the voids in the eggshell structure. (E) All-Euler angle maps showing the relative orientation of crystalline domains. Note that cements retain the orientation of the crystals they are attached to, growing in epitaxy. Arrows in B point to the location of extra-spherulites, with white arrows pointing to longitudinally cut extra-spherulites and the black arrow pointing to a transversally sectioned extra-spherulite. Location of Fig 6 details is shown in C.

**Fig 5:**
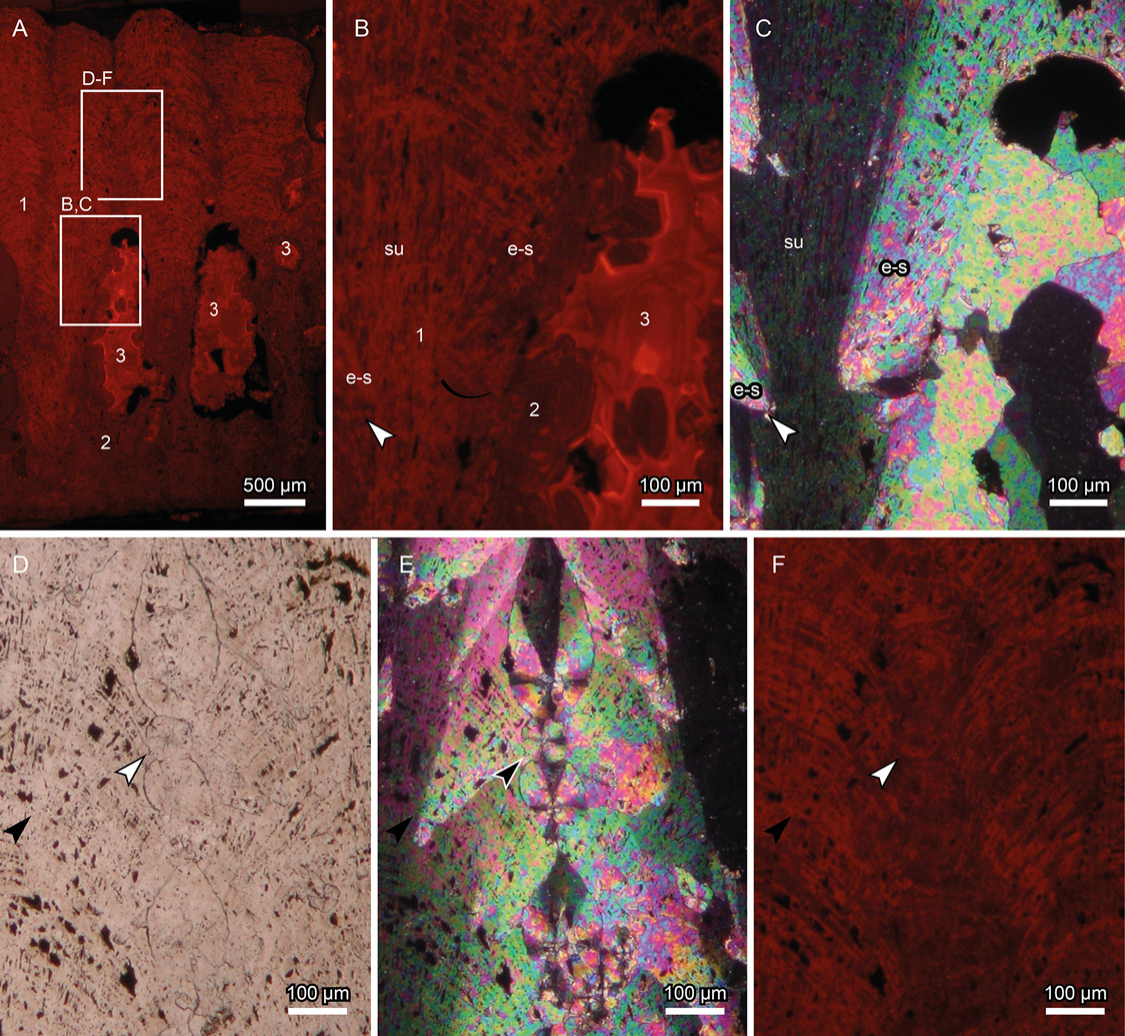
*Megaloolithus* cf. *siruguei*, PS-TEC,215, radial thin section. Cathodoluminescence (A, B and F), and transmitted light images (cross polarized, E, parallel polarized, D). (A) Full eggshell section, showing that the entire eggshell presents orange luminescence, implying that the original composition of the eggshell has been altered. Note that three shades of orange can be distinguished; these are interpreted as three consecutive recrystallization stages, and precipitated under different redox conditions. Numbers (also in B) indicate the proposed temporal succession of these stages. Squares indicate the location of detailed images. (B,C) Detail of extra-spherulites (e-s) shown in B, which grow in an angular relation to the shell unit (su). Note the lack of organic cores or equivalent structures at the base of the extra-spherulites. The extra-spherulites show the same luminescence as the shell units, dating the growth of these structures between eggshell formation and the recrystallization of the lower part of the eggshell and the growth of the drusy cement. A second recrystallization event obliterates the structure of the bottom and middle part of the eggshell, represented by the darker luminescence. At this point, drusy cements start to fill the voids in the eggshell structure. Area 2 and area 3 cements precipitate in a syntaxial relation, and there is no evidence to establish whether they are two different cements or two phases of the same cement’s redox evolution, which becomes progressively richer in luminescence activator cations or depleted of luminescence quencher cations. (D-F) Details of A, where three transversally sectioned extra-spherulites can be seen, surrounded by longitudinally sectioned ones. Note the voids surrounding the extra-spherulites, pointing towards a dissolution-precipitation origin. Arrow points towards a spherulite that shows a slightly different luminescence outline, supporting the diagenetic origin of these structures.

PS-TEC,215 presents the typical features of Megaloolithidae under polarized light. The shell units are tall and slender, with a height to width ratio of 1:7, and are isolated, with no signs of fusion throughout the eggshell thickness (Fig 4A). Several interstices appear between shell units, filled with spar calcite cement. The contour of these interstices is irregular, suggesting that at least partial dissolution of the shell units occurred. The interstices do not cross the whole eggshell up to the outer surface, and may represent either dissolution voids or be part of the pore system, which seems to be slightly branching or at least oblique, as reported in other *M*. *siruguei* eggshells [37]. Incipient shell units, which do not reach the half-thickness of the eggshell, are also present in the lower part of the section, but it is not possible to exclude the possibility that they may be artifacts of thin section preparation. Faint growth lines can be observed in plane polarized light and are present through the whole eggshell, but they are subtle and concave with respect to the growth direction (Fig 4A). They are brownish in color, and present a major concentration of pits, thus pointing to the presence of organic matter not preserved in the fossilized specimen. Additionally, they do not appear to be crossing boundaries between shell units, although this may be the result of intense alteration of the eggshell in the contact between shell units (see below and Fig 5A and 5F). Most mammillary cones are eroded, but some of them are partially preserved. Under cross polarized light, several interesting and surprising features can be observed (Fig 4B). The general aspect of the eggshell unit is that of other Megaloolithidae eggshells, with units showing undulating extinction. Nonetheless, in the proximity of the boundaries between shell units several extra-spherulites can be observed, resembling the mammillary cones of the inner surface of the eggshell in their fanning structure. These extra-spherulites measure less than 500 μm in mean length on the analyzed surface, and, on this same surface, they grow obliquely to the general direction of eggshell growth, at an angle of around 20 to 30 degrees to it (Fig 4A and 4C). Other extra-spherulites, located at the very boundaries between shell units, grow towards (or away from) the section surface, resulting in sections of radial aggregates of calcite crystals. Interstices between the shell units show irregular contours, suggesting that some degree of dissolution of the adjacent shell units has taken place. In addition, the interstices show calcite cement infilling, formed after the growth of the extra-spherulites (see discussion), in an apparently syntaxial relationship with the eggshell, with well-formed euhedral crystals. The crystal grain size increases in the middle of the interstices, which suggests that they were precipitated from a fluid that saturated the interstices, forming a drusy mosaic cement. All the three units observed in the eggshell area analyzed show a partial to complete replacement by euhedral crystals at the level of the interstices, suggesting that the cement grew over the shell units, obliterating the eggshell ultrastructure.

### Cathodoluminescence

PS-TEC,215 displays a bright orange luminescence under standard analysis conditions (see materials and methods section; Fig 5A), thus indicating an intense modification of the original low-magnesium CaCO_3_ composition of the eggshell. Luminescence is more intense following crystalline defects (i.e. natural interruptions of the crystalline symmetry). For example, growth lines are highlighted, probably due to the accumulation of luminescence-activating elements in the voids left by the former organic matter. The boundaries between individual crystalline domains, where defects accumulate, also show bright luminescence. Noticeable is that even in areas between these bright orange luminescent lines, there is a darker shade of orange luminescence, suggesting that there is no relict of the original composition of the eggshell (Fig 5).

Three different zones of luminescence can be differentiated in the sample, and are numbered accordingly in Fig 5: 1) a moderately bright area, comprising most of the upper part of the eggshell, apart from the areas between adjacent shell units; this area is where all of the extra-spherulites are located; 2) a less bright area comprising the lower and middle part of the eggshell and the areas between adjacent shell units, as well as part of the secondary drusy cements that fill the voids in the eggshell; 3) the brightest area, which is restricted to the final growing stages of the aforementioned drusy cements.

The growth zones of the drusy cement are thus revealed by different shades of orange luminescence (Fig 5B and 5C). Two different episodes of crystal growth can be differentiated. The first stage is mainly dark orange, with a few thin bands of a brighter shade of orange luminescence. The grains formed at this stage are mainly attached to the interstice boundaries, but a few detached grains also grow at this stage. This phase shows smooth contacts with the darker phase of the shell units, and shows the same luminescence; these two stages thus probably formed at the same time. The second cement stage (labeled 3 in Fig 5) presents much brighter colors, with the darkest shade of orange being considerably more luminescent than the lighter bands of the previous stage. This cement fills the gaps between the previous grains, and grows syntaxially on the previous cement (Fig 5C).

Extra-spherulites are thus restricted to the areas of the eggshell in area 1 of luminescence. Noticeable is that the extra-spherulites cannot easily be distinguished under cathodoluminescence (Fig 5B), suggesting that they were formed in the same conditions as the rest of the area. Nevertheless, the boundaries of some of these structures are faintly highlighted by the luminescence (white arrow in Fig 5B and 5C). This is more evident in the extra-spherulites that grow perpendicular to the analyzed surface. The slight increase in luminescence matches small voids that surround these structures (Fig 5D–5F), pointing towards a secondary origin, even though not all of these voids can be highlighted in the cathodoluminescence pictures. Thus, the hypothesis of a primary origin for the extra-spherulites can be rejected.

### Electron backscatter diffraction

**Fig 6:**
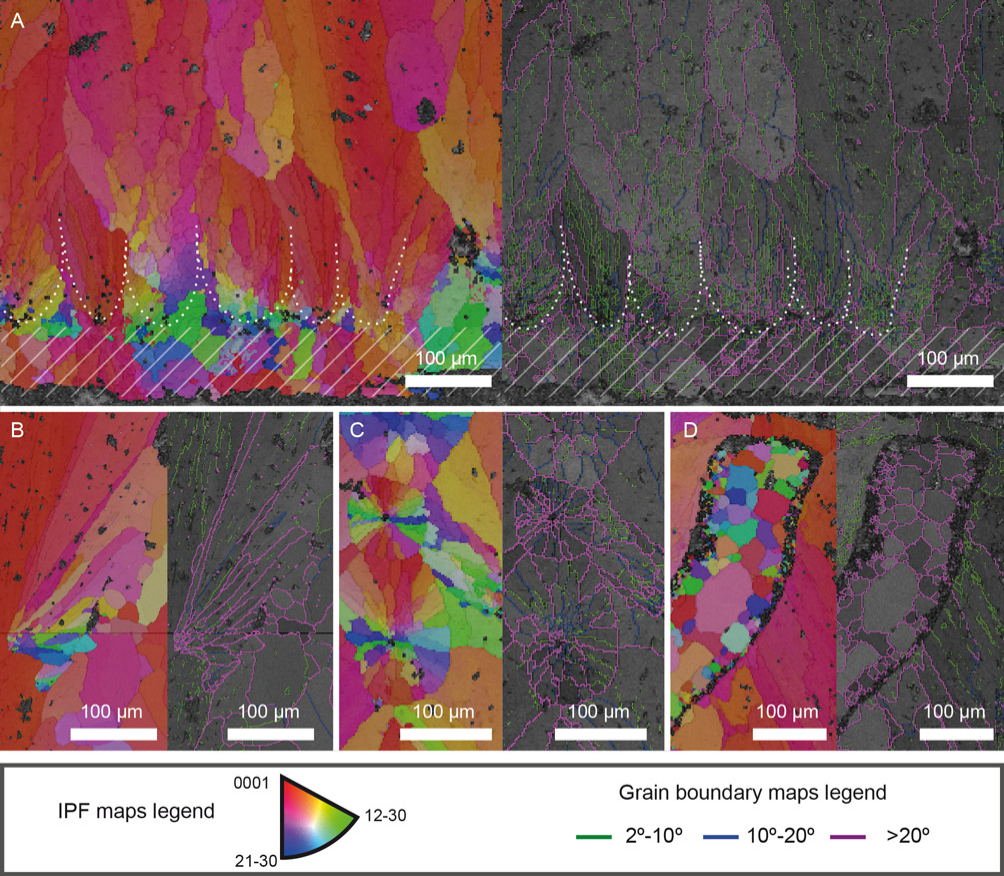
*Megaloolithus* cf. *siruguei*, PS-TEC,215, radial thin section. Comparison between true mammillae and extra-spherulites. Each sub-figure shows the inverse pole figure coloring maps on the left and the grain boundary maps over a band contrast image on the right. (A) Detail of the mammillae at the base of the shell units. Dashed area denotes the spar cement that covers the inner surface. White dotted lines delineate the approximated limits of different mammillae. Note the rapid reorientation of crystals due to competitive growth (see [2] for details on this mechanism of eggshell formation in fossil dinosaur eggshells). The mammillae show a profusion of low-angle boundaries. (B) Detail of a longitudinally sectioned extra-spherulite. Note that it grows with its main axis oblique to the direction of eggshell growth and that most grain boundaries are high-angle boundaries (over 20°). Note that the orientation of the large red crystalline domain that comprises the shell unit on the left is not affected by the presence of the extra-spherulite, suggesting that the secondary shell unit was formed with the eggshell fully formed. (C) Detail of two extra-spherulites transversally sectioned, which grow towards or out from the thin section surface. Again, the lack of low-angle boundaries contrasts with their abundance in the shell units. (D) Detail of a void, probably part of the eggshell pore system, where spar cement has grown. Note the typical increase in grain size from the boundaries of the void to the center. Note the lack of low-angle boundaries.

Inverse pole figure color-coded maps, plotted in the direction of the y-axis (vertical) (IPF; Figs 4C and 6), all-Euler orientation maps (Fig 4E), and grain boundary maps overlying band contrast images (Fig 4D) were used to analyze orientation and misorientation data obtained using EBSD. The general crystallographic architecture of the eggshell resembles that of other dinosaur eggshells, with elongated calcite crystals that grow with their c-axis parallel to the direction of eggshell growth [2]. Noticeable is that, although intense recrystallization of the eggshell has occurred, as evidenced by the cathodoluminescence analysis, for most of the sample examined, the general orientation of the crystallographic domains of the eggshell is what would be expected of a fine preserved eggshell. An important exception to this is the area between the voids shown in Fig 4. In this area, the structure and crystallographic texture of the eggshell has been completely obliterated by the calcitic cement (Fig 4B–4E), replacing the radial crystal pattern with a drusy cement, identical to that which fills the voids in the shell units. Furthermore, in this area the growth lines are not preserved.

The innermost part of the eggshell shows a wide range of orientations, suggesting radial growth during the first stages of the eggshell mineralization. Nevertheless, level with the system of interstitials, most crystals already present a low angle of the c-axis in relation to the direction of growth. This has previously been postulated as evidence that competitive growth is the mechanism that controls eggshell formation in dinosaurs [2]. In *M*. cf. *siruguei* this competition occurs at several levels, as there is competition between crystals of the same shell unit, which results in the preferred orientation of the eggshell structure. But there is also competition between shell units, which results in a lack of space in the outer part of the eggshell that causes the abortion of some of the shell units that grow from the mammillae, in turn resulting in the complex pore system previously mentioned. It is noticeable that, in the inner part of the eggshell, some areas, especially where the organic cores are located, yield no EBSP. This has been reported in other dinosaur eggshells [2] and may be due to the relative abundance of organic material in the organic cores and the mammillary cones, as well as to material loss during sample handling and preparation. Also, a thin layer (<100 μm thick) of spar cement covers the inner surface of the eggshell (dashed area in Fig 6A).

Particularly interesting is the crystal orientation in the extra-spherulites that grow in the proximity of the boundaries between the shell units (Fig 6B and 6C). As noted above, these units grow at an angle of 20 to 30 degrees with respect to the predominant growth direction. This results in a peculiar disposition of the crystals. Those crystals closer to the middle of the shell unit present c-axis orientations that are close to the general direction of eggshell growth, whereas those crystals on the other margin of the extra-spherulites present misorientation of over 45 degrees with respect to the eggshell growth direction.

Grain boundary maps were obtained for 2°-10°, 10°-20° and >20° misorientation angles (Fig 4D). As in other dinosaur eggshells, the inner part of the eggshell presents an abundant number of low-angle boundaries, growing inside small grains surrounded by high-angle boundaries. As the eggshell grows, fewer boundaries appear, with most of the eggshell consisting of a few crystals delimited by high-angle boundaries with several sub-parallel low-angle boundaries inside, which reflects the small misorientation angles that give the dinosaur eggshells their characteristic undulating extinction. Drusy cements that fill the interstices between shell units present high-angle boundaries (most of them over 35°; Fig 6D).

The extra-spherulites again present the most striking features in the grain boundary map (Fig 6B and 6C). All of them are misoriented with respect to the shell unit, showing boundaries of over 20°. Even those grains that shared the orientation of the c-axis in the IPF coloring map show high misorientation angles, suggesting that crystal axes a and b are not oriented with the eggshell growth direction even when the c-axis is. Furthermore, these extra-spherulites present very few low-angle boundaries, and are usually formed by up to fifteen crystals with over 20° misorientations (Fig 6B and 6C). They contrast particularly with the true shell units, where, as mentioned above, low-angle misorientation boundaries are more abundant (Fig 6A).

All-Euler maps confirm the syntaxial growth of the cements infilling the voids observed in the cross polarized light and cathodoluminescence images (Fig 4E), as they maintain the same crystallographic orientation as the crystal that they have overgrown.

Also, there is no evidence of significant misorientation in the contact between the areas of the shell units with different luminescence in the left shell unit (compare Fig 4C with 5A), which implies either that crystal growth is continuous during the two stages or that in the final stage the growth is always in a perfectly syntaxial relationship.

## Discussion

### Genesis of the extra-spherulites

Cathodoluminescence analyses allow us to postulate a model for the evolution of the crystallography in eggshell taphonomic history. The overall luminescence of the eggshell implies that the original mineralogical composition has been lost, despite the preservation of ultrastructural and histostructural features of the eggshell, such as former organic cores and growth lines. Furthermore, in most of the eggshell the only alterations of the original crystallographic texture are the extra-spherulites, although other areas have been completely replaced.

The taphonomic history of the eggshell can be reconstructed as the following succession of events:

An original non-luminescent eggshell was buried, and exposed to different fluids with different redox conditions during fossil diagenesis. The first fluid recrystallizing the eggshell replaced all its composition, but preserved the texture in high detail, thus pointing to a very slow, atom-by-atom replacement of the mineralogical composition. The only anomalous structures observed are the extra-spherulites. An undetermined amount of time afterwards, a second phase of recrystallization occurs. This new fluid is either richer in cathodoluminescence quencher units or more pure in its CaCO_3_ composition, as its luminescence is lower. This fluid partially obliterates the structure of the eggshell, and forms the first preserved cement in the infilling gaps, either replacing a previous infilling completely or filling the interstices for the first time. This supports a secondary origin for the interstices. Finally, a third cement grows and fills the interstices, frequently over the previous cement 2. Given the available data, it is impossible to establish whether cement 2 and cement 3 grow from different fluids, or whether the change in luminescence is the result of the evolution of a single fluid with a variable composition. Thus, the three fluids precipitated under different conditions, as evidenced by the textural relations between them, with fluid 1 preserving the original eggshell structure whereas fluid 2 at least partially replaces it with euhedral crystals. It is important to emphasize that there is no evidence of the absolute timing of these processes, and all the processes are only relatively dated.

PS-TEC,215 shows a similar crystallographic architecture to other megaloolithid eggshells, with tall shell units with tabular calcite crystals with a sub-vertical c-axis, fanning out from the mammillary cones at close angles, but additionally presenting abundant extra-spherulites. The extra-spherulites present a c-axis that is not parallel to the direction of eggshell growth. They present several features that point towards a secondary origin. First, the random distribution of the extra-spherulites in such an otherwise highly hierarchical structure as an eggshell is suspicious, specifically when the number and development of these units varies between samples. Secondly, extra-spherulites are restricted to the boundaries of the true shell units, growing in the interphase of the shell unit and the interstices or adjacent shell units, all of them locations which are more exposed to dissolution processes (Fig 4B, 4D, 4E and 4F). Third, IPF coloring maps (Figs 4C, 5B and 5C) show that most of the extra-spherulites grow at an angle to the general direction of eggshell growth, but in a close angular relation with the neighboring crystals, without disturbing the growth of the crystals that build the true shell units. Fourth, extra-spherulites show high misorientation boundaries with neighboring units, either primary or secondary ones. There are misorientation angles over 20° even in the cases where the secondary shell unit crystals grow with their c-axis parallel to the eggshell growth (Figs 4B, 4E, 6B and 6C). Fifth, extra-spherulites lack organic cores, by contrast with true shell units. Finally, the lower number of low-angle boundaries inside the extra-spherulites suggests that these crystals grow with fewer crystallographic defects, pointing to a slower crystallization than the true shell units. This can also be observed in optical microscope images, where the extra-spherulites show fewer inclusions of organic matter within their structure.

It has been postulated that the presence of protein relicts in the shell units may result in the nucleation of inorganic carbonate aggregates that, when fully grown, may be indistinguishable from true shell units under the optical microscope [11]. Proteins present in the eggshell membrane are known to control the crystallographic growth of the eggshell, and inorganic calcite dramatically changes its crystal morphology when exposed to different uterine and eggshell proteins [4]. Thus, it is coherent that secondary calcite deposits may have shell-unit-like crystal habits in the presence of these proteins. Most proteins are known to decay rapidly during fossilization, but eggshells have proven to be a good environment for protein preservation, as most of the organic matter is intracrystalline [38]. Thus, protein control of the nucleation and growth of the extra-spherulites cannot be ruled out, especially if the recrystallization and precipitation of phase 1 took place at a very early stage of diagenesis. In this scenario, previous dissolution of the eggshell is necessary to allow undisrupted growth of the extra-spherulites. As shown in Fig 5D–5F, some of the extra-spherulites show evidence of previous dissolution of the surrounding crystals, but this is not the rule.

Another scenario can be postulated for a secondary origin of the extra-spherulites. If calcite was replaced in an atom-by-atom process, it is possible that at least some of the low-angle crystallographic defects of the calcite were not replicated. As a result of the replacement of the calcite acicular fans of the original eggshell, this process would result in larger crystalline domains with lower-angle boundaries within them, and with higher-angle relations with their neighbors. A similar process has been described in fossil bone, where the replacement of the hydroxyapatite that constitutes the living form with the more stable phase of fluorapatite results in a significant increase in domain size, but without altering the shape of the crystals forming the fossil [39,40]. This process requires neither the previous dissolution of the eggshell nor exceptional preservation of the proteins, thus being more plausible.

### Implications for the fossil record of secondary shell units

Secondary shell units have been described in most of the oofamilies of dinosaur fossil eggshells [7,9,10]. Aborted shell units are frequent in megaloolithid eggshells [12,37]. Double-layered eggshells are also frequent, with two different variants: isolated shell units that grow on top of true eggshell units, sometimes without organic cores [7,9]; and true multilayered eggshells, where continuous, well-formed layers of new eggshell units develop over a previous complete eggshell, sometimes with evidence of newly formed *membrana testacea* between the layers [7]. Finally, extra-spherulites have been previously described in Megaloolithidae eggshells [12].

The secondary origin of extra-spherulites reinforces the need for detailed taphonomical analysis prior to the description of eggshell structures as biological or pathological features. Recent works have described several new taxa on the basis of the presence of secondary shell units. The case of PS-TEC,215 is somewhat similar to what is seen in some Chinese oogenera belonging to Favoloolithidae, Spheroolithidae and Dyctiolithidae [6,9], in that the secondary shell units grow at different heights within the eggshell, but it shows some important differences, such as the fact that the extra-spherulite units do not abut with adjacent ones and never form full secondary layers. Nevertheless, our results show that caution is needed when regarding secondary shell units as diagnostic parataxonomical characters, at least when considering the particular case of extra-spherulites, as they may form under variable diagenetic conditions. Our evidence supports the objections presented by other authors [16,18] on the nature of the secondary shell units in the Chinese material, which needs a deep revision before the complex mechanisms of eggshell formation proposed by [8,10] can be accepted. We suggest that EBSD, combined with cathodoluminescence and SEM observations, may help to elucidate this controversy.

An example of the complexity of studying taphonomical alterations can be found in [7] These authors studied the presence of abnormal shell units and eggshell layers in titanosaur eggs, and in a subsequent study [17] proposed a list of criteria by which to identify truly pathological eggshells, in order of decreasing reliability: presence of permineralized membrana testacea at the bottom of secondary shell units; conformation of secondary shell units over unweathered ornamentation of true shell units; structural relationships (e.g. pore truncation, smooth contacts between shell units, and optical continuity). Other authors [11] discuss these results and add some interesting taphonomical observations, suggesting that several of the pathological eggshells recognized by [7] at the classical Auca Mahuevo site (Late Cretaceous, Argentina) may have been taphonomical artifacts, and proposing the combined use of cathodoluminescence (CL), backscattered scanning electron microscopy (BSE) and energy-dispersive X-ray spectroscopy (EDS) to elucidate a possible diagenetic origin of the secondary shell units. Although CL has been confirmed to be useful in identifying authigenic calcite cements in fossil eggshells ([34]; this work), we consider that the analysis carried out by [11] is not conclusive concerning the diagenetic origin of these secondary shell units. The alleged evidence for a secondary origin is based on the presence of luminescence in the contacts of the secondary shell unit with the rest of the eggshell and surrounding dissolution voids in the interstices between true and secondary shell units [11], but it is noticeable that the purported secondary shell unit is itself non-luminescent, contrary to what is seen in the specimens studied in this work. The presence of luminescence in the contacts between shell units is not evidence of a secondary origin, as these discontinuities are more unstable and may be more easily diluted and re-precipitated, and do not imply that these shell units are authigenic. On the other hand, the dissolution voids dissolve both the shell units and the extra-spherulites, this being evidence that the luminescent infilling is posterior to the secondary shell unit formation. Cathodoluminescence is based on eggshells being formed in a non-luminescent form of calcite, due to the lack of trace elements that show luminescence, but diagenetic cements are usually enriched in elements that do emit high luminescence under CL (usually Mn2+), and this is widely used to recognize these cements.Thus, [11] make important progress with respect to previous studies on CL applied to eggshell, ruling out the presence of elements that may quench the luminescence of calcite (usually Fe2+) with the use of EDS and BSE, but a third, more complex scenario is omitted: if the authigenic calcite formed in the eggshell is not enriched in any of these elements (pure calcite), CL, BSE and EDS will fail to identify the cements as taphonomical artifacts. Here we propose that detailed analysis of the crystallographic structure of these secondary shell units via EBSD, searching for features observed in our samples, such as an anomalous lack of low-angle boundaries, or an unexpected increase in crystalline domain size, may be the only way to resolve this complex problem. This approach has already been successfully applied in a recent study [41], where EBSD was applied to fossil eggshells prior to conducting isotopic analyses, in order to discard altered samples that look unaltered when examined under SEM and optical microscopy.

## Conclusions

The generalized orange luminescence observed in PS-TEC,215 suggests that the eggshell fragment has been completely recrystallized, and that the original composition of the eggshell has not been preserved. At least two different recrystallization events have occurred, resulting in three differently luminescent calcite phases. The first recrystallization event resulted in the formation of extra-spherulites, which resemble small shell units growing at the limits of true shell units.

Electron backscattered diffraction has been used to analyze the detailed ultrastructure of these extra-spherulites and of the cements infilling several eggshell interstices. Inverse pole figure coloring maps show that there is a high-angle crystallographic relation between the extra-spherulites and the shell units of the eggshell, although the crystallography of the true shell unit is not disturbed by these secondary shell units. Grain boundary maps show that high-angle misorientation boundaries isolate the secondary shell units from the true shell units, and that the number of low-angle boundaries (i.e. crystalline defects) is reduced in the secondary shell units compared with the true ones. These features are also shown in the diagenetic drusy cements filling the pore system of the eggshell. All this evidence supports a diagenetic origin for these extra-spherulites.

The presence of secondary shell units has been used in the past both to describe new parataxa and identify pathologies in fossil eggshell. Our study shows that these features may have a taphonomical origin, and should be studied in detail before any parataxonomical or ecological conclusions are drawn. EBSD augments other microscope techniques (CL, EDS and BSE) as a useful tool for understanding taphonomical alterations in fossil eggshells.

## Supporting Information

S1 File Raw EBSD data of PS-TEC,215. CPR and CRC Channel 5 files containing the acquired EBSD data, after the combination of the 27 acquired maps.
(ZIP)
